# ClueNet: Clustering a temporal network based on topological similarity rather than denseness

**DOI:** 10.1371/journal.pone.0195993

**Published:** 2018-05-08

**Authors:** Joseph Crawford, Tijana Milenković

**Affiliations:** 1 Department of Computer Science and Engineering, University of Notre Dame, Notre Dame, IN, United States of America; 2 ECK Institute of Global Health, University of Notre Dame, Notre Dame, IN, United States of America; 3 Interdisciplinary Center for Network Science and Applications (iCeNSA), University of Notre Dame, Notre Dame, IN, United States of America; Tokyo Institute of Technology, JAPAN

## Abstract

Network clustering is a very popular topic in the network science field. Its goal is to divide (partition) the network into groups (clusters or communities) of “topologically related” nodes, where the resulting topology-based clusters are expected to “correlate” well with node label information, i.e., metadata, such as cellular functions of genes/proteins in biological networks, or age or gender of people in social networks. Even for static data, the problem of network clustering is complex. For dynamic data, the problem is even more complex, due to an additional dimension of the data—their temporal (evolving) nature. Since the problem is computationally intractable, heuristic approaches need to be sought. Existing approaches for dynamic network clustering (DNC) have drawbacks. First, they assume that nodes should be in the same cluster if they are densely interconnected within the network. We hypothesize that in some applications, it might be of interest to cluster nodes that are topologically similar to each other instead of or in addition to requiring the nodes to be densely interconnected. Second, they ignore temporal information in their early steps, and when they do consider this information later on, they do so implicitly. We hypothesize that capturing temporal information earlier in the clustering process and doing so explicitly will improve results. We test these two hypotheses via our new approach called ClueNet. We evaluate ClueNet against six existing DNC methods on both social networks capturing evolving interactions between individuals (such as interactions between students in a high school) and biological networks capturing interactions between biomolecules in the cell at different ages. We find that ClueNet is superior in over 83% of all evaluation tests. As more real-world dynamic data are becoming available, DNC and thus ClueNet will only continue to gain importance.

## Introduction

Networks (or graphs) can be used to model complex real-world systems in a variety of domains. Examples include technological systems such as the Internet or power grids, information systems such as the World Wide Web, social systems such as Facebook or real-life friendships, ecological systems such as food webs, or biological systems such as the brain or cell [1, 2]. There exist many computational strategies for network analysis, each answering a different applied question. One of the popular computational strategies is network clustering (also known as community detection). In particular, we focus on clustering of a dynamic network as opposed to traditional clustering of a static network.

In general, the goal of network clustering is to divide the network into groups (i.e., clusters or communities) of “topologically related” nodes. The resulting topology-based clusters are expected to “correlate” well with node label information, i.e., metadata. Examples of metadata are cellular functions of genes/proteins in biological networks, or age or gender of people in social networks.

Even for static data, the problem of network clustering is complex. First, while often “topologically related” means “densely interconnected”, “topologically related” *can* mean different things. Other notions of topological relatedness *have* been used [3–5], partly because dense interconnectedness does not always “correlate” well with node metadata [6, 7]. That is, the notion of a cluster is not precisely defined. Second, the problem is computationally intractable (i.e., NP-hard) [8]. Hence, heuristic approaches for network clustering need to be sought. Third, these approaches typically have a number of parameters, and it is often difficult to determine their optimal values. All of these factors contribute to the complexity of the network clustering problem for static data. For dynamic data, the problem is even more complex, due to an additional dimension of the data, namely their temporal (evolving) nature. This not only makes the definition of a dynamic network cluster more complicated, but it also adds to the computational complexity of the problem, including a likely increase in the number of method parameters. So, further problem formulations and methodological solutions are needed in the context of dynamic network clustering (DNC). This is exactly the focus of our study.

**Fig 1:**
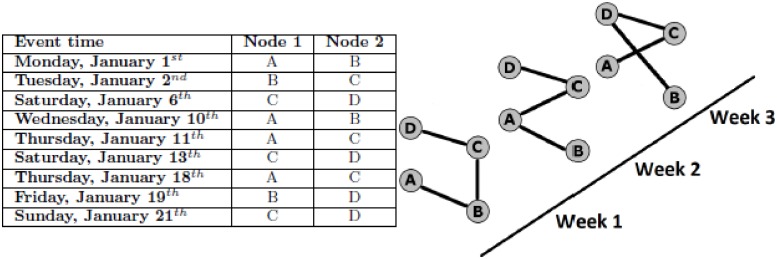
Illustration of how a raw temporal dataset (left) is modeled as a dynamic network (right). One parameter is the length of the temporal window during which interactions are aggregated. In our illustration, this parameter value is one week (note that weeks begin on Monday and end on Sunday). For example, the network snapshot for week 1 (January 1^*st*^ through January 7^*th*^) will aggregate interactions between nodes A and B, B and C, and C and D. Another parameter is the minimum number of events that must occur between the same nodes within the given time window in order to link these nodes in the corresponding snapshot of the dynamic network. This parameter is set to one in this example.

Before we can further discuss the topic of DNC, we need to comment on how a temporal dataset is modeled as a dynamic network. A temporal dataset contains information on when each temporal interaction (i.e., event) occurs and between which elements. Such a dataset is typically represented as a series of network *snapshots*, where each snapshot aggregates the temporal data (i.e., all events) observed during the corresponding time interval (see Fig 1 for an illustration and Section Definitions for a formal definition).

There exist two general categories of approaches for DNC, each with its own (dis)advantages: snapshot clustering [9] and consensus clustering [10]. On the one hand, snapshot clustering finds a separate partition (i.e., division of a network into clusters) for each network snapshot. Given the snapshot-level partitions, this type of clustering can be used to track the evolution of the network by matching individual clusters in adjacent snapshots [11–14]. However, snapshot clustering partitions each snapshot in isolation from all other snapshots. As such, it disregards any inter-snapshot relationships in the dynamic network, which capture valuable temporal information from the underlying data. On the other hand, consensus clustering finds a single partition for the entire network that fits reasonably well all of the snapshots. Thus, this type of clustering takes into consideration the evolution of the network as a whole, even though because of this, consensus clustering loses more detailed, snapshot-specific information. Yet, because consensus clustering accounts for more temporal information than snapshot clustering, and because of recent popularity of consensus clustering (e.g., it can be used to cluster not just dynamic but instead heterogeneous data [15]), we focus on this clustering type. Henceforth, we refer to consensus clustering simply as DNC. Prominent existing approaches of this type are: Louvain [16], Infomap [17], Hierarchical Infomap [18], label propagation [19], simulated annealing [20], and MultiStep [21]. The existing approaches have two major drawbacks, which we aim to address in this study, as follows.

First, all of the existing approaches assume that nodes should be in the same cluster if they are densely interconnected within the network. This common notion of nodes being topologically related if they are densely interconnected with each other is known as the *structural equivalence* [1]. In some domains and applications, it might be of interest to group (cluster) nodes that are topologically similar to each other instead of or in addition to requiring the nodes to be densely interconnected. This notion of nodes being topologically related if they are topologically similar is known as the *regular equivalence* [1]. We hypothesize that clustering based on nodes’ topological similarity instead of or in addition to their interconnectivity denseness will result in a higher-quality partition, and we propose an approach for achieving this (see below).

Second, all of the above approaches except MultiStep were originally designed for solving the static network clustering problem and were later adjusted to the problem of DNC. Possibly because of their original intention, even when using their adjusted versions on dynamic network data, they still tend to ignore some of the valuable temporal information in the dynamic data. Specifically, this is because they generate a partition of the given dynamic network as follows. These methods are run on each snapshot, in order to produce one partition per snapshot. Then, they aim to combine the snapshot-level partitions into a single network-level consensus partition by relying on the concept that if two nodes *i* and *j* appear in the same cluster in many of the snapshot-level partitions, then *i* and *j* should have a high chance of being in the same cluster in the consensus partition. Specifically, *i* and *j* will have a high similarity score if they appear in the same cluster in many of the snapshot-level partitions, and a low similarity score otherwise. The resulting pairwise node similarity scores are stored in what is called a consensus matrix, which existing methods use to generate the consensus partition (see Section Existing methods and S3 Section for more details about this process). Clearly, the first and major step of the existing consensus DNC approaches (prior to constructing the consensus matrix) is simply snapshot clustering as defined above. Thus, these approaches inherit the key problem of snapshot clustering—they ignore any inter-snapshot relationships in their first step, and they only implicitly consider them when they combine the individual snapshot-level partitions into the consensus partition. We hypothesize that capturing these relationships explicitly, i.e., earlier in the process, prior to performing any clustering, will result in a higher-quality partition, and we propose an approach for achieving this (see below).

**Fig 2:**
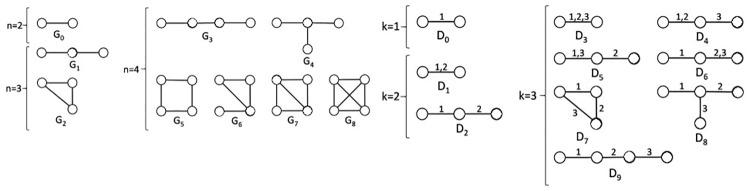
Illustration of (a) static and (b) dynamic graphlets. (a) All nine static graphlets with up to four nodes. (b) All dynamic graphlets with up to three events. Multiple events along the same edge are separated with commas. We note that only smaller graphlets are shown for both static and dynamic graphlets for the purpose of illustration, but larger graphlets are used. The figure originates from [5].

To test both of the above hypotheses, we need to introduce a novel consensus clustering approach that can both: 1) cluster nodes that are topologically similar in the evolving network, and 2) capture inter-snapshot relationships (and thus as much temporal information as possible) very early in the process of constructing a consensus partition. With these two tasks in mind, we explore the notion of graphlets [3]. Graphlets (in the static setting) are small connected subgraphs of a network, such as a cycle (e.g., triangle or square), a path, a clique, or any other small network structure (Fig 2(a)) [3]. Graphlets were used for a number of computational [22, 23] and applied [24–27] tasks in the static setting. But, they can be adjusted to the dynamic setting as well, as follows.

Given the notion of static graphlets, for each snapshot of a dynamic network, we are able to count how many times the given node participates in the given static graphlet. By doing this for each graphlet, we can form the node’s graphlet degree vector (GDV), which quantifies the information about the *extended* neighborhood of the node in the given snapshot of the dynamic network [3]. To summarize the node’s position in the entire dynamic network, we can gather its GDVs for all snapshots and concatenate these, which results in what we refer to as the static-temporal GDV (ST-GDV) of the node in the dynamic network. Given ST-GDV information for each node in the dynamic network, by comparing the nodes’ ST-GDVs, we obtain a measure of similarity between the nodes’ evolving network neighborhoods. Then, we can feed the resulting node similarities into any clustering algorithm to get a single (implicit consensus) partition for the whole dynamic network. As a proof of concept, we mainly use the *k*-medoids algorithm.

This approach that is based on static graphlets helps us address the first issue with the existing methods: clustering nodes based on topological similarity rather than denseness. However, even this approach considers each network snapshot in isolation when computing snapshot-level GDVs. Thus, it also ignores valuable inter-snapshot relationships, just like the existing denseness-based methods do. To address this, we rely on a recent notion of dynamic graphlets, which is an extension of static graphlets to the dynamic setting [5]. This extension is done by assigning temporal information to the edges, which thus become events (see Fig 2(b) for an illustration, and Section Definitions and S1 Section for a formal definition).

**Fig 3:**
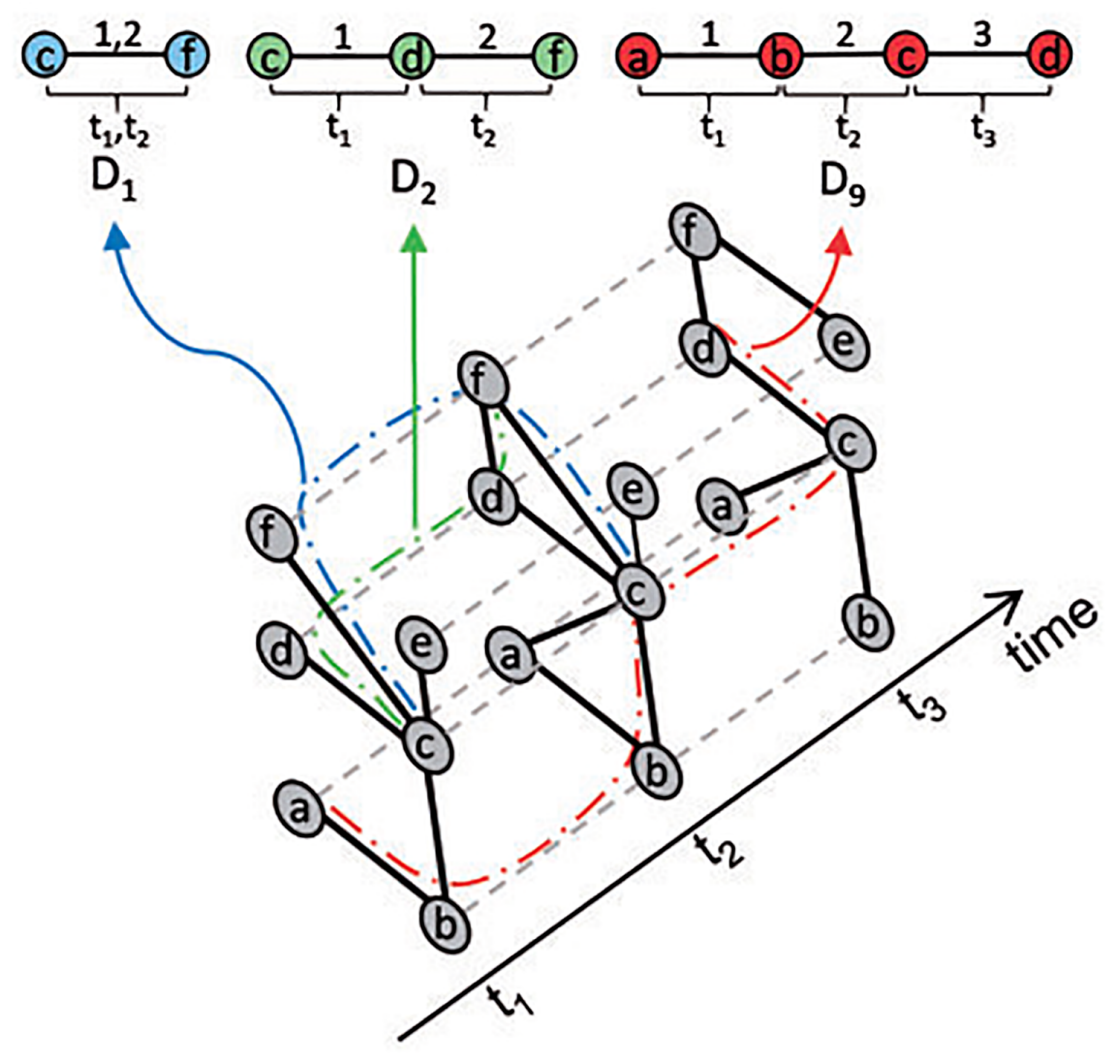
Illustration of the existence of dynamic graphlets *D*_1_, *D*_2_, and *D*_9_ in a toy dynamic network with three snapshots. Dashed lines denote instances of the same node in different snapshots. Colored lines denote what it means for *D*_1_ (blue), *D*_2_ (green), and *D*_9_ (red) to exist in a network. The figure originates from [5].

Given the notion of dynamic graphlets, for each node in a dynamic network, we are able to count how many times the given node participates in each dynamic graphlet, which results in the node’s dynamic GDV (D-GDV; Fig 3), which now quantifies the information about the extended neighborhood of the node in the entire dynamic network (rather than in only one of its snapshots). Then, just as with ST-GDVs, we can compute pairwise node similarities by comparing the nodes’ D-GDVs and feed these into any (e.g., *k*-medoids) clustering algorithm.

The advantage of dynamic graphlets (D-GDVs) over static ones (ST-GDVs) is that a dynamic graphlet spans multiple snapshots and thus captures inter-snapshot relationships, while a static graphlet captures intra-snapshot relationships. Hence, dynamic graphlets can address both drawbacks of the existing methods, while static graphlets can address only the first one. Therefore, we expect that using dynamic graphlets will result in higher-quality clusters than using static graphlets. We evaluate in this study whether this expectation holds in practice.

**Fig 4:**
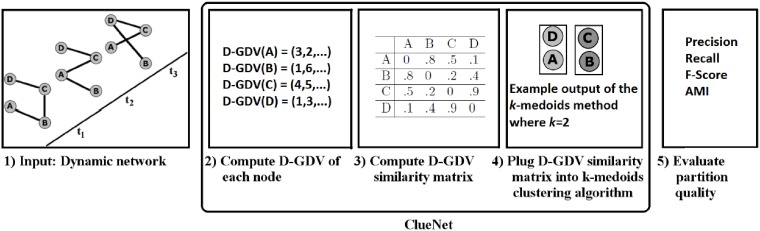
Summary of ClueNet.

The entire pipeline for clustering a temporal network using graphlets as described above is our new DNC approach, which we call ClueNet (C). We have three versions of ClueNet. One version is based on static graphlets (C-ST), another one is based on dynamic graphlets (C-D), and the remaining one is based on “constrained” dynamic graphlets (C-C). The latter intuitively is a more restrictive and thus faster version of dynamic graphlets [5]. We test constrained dynamic graphlets because dynamic graphlets can be time-consuming on large networks. So, we are interested to see whether we can obtain a speed-up over dynamic graphlets without sacrificing accuracy. Note that as long as at least one of C-ST, C-D, or C-C is superior to the existing methods, this would justify the need for our ClueNet approach. The steps of the approach and our study are summarized in Fig 4 (using C-D as a representative version of ClueNet).

We evaluate our three versions of ClueNet against the six existing DNC methods mentioned above and described in Section Existing methods, S3 and S4 Sections. We do so on both social networks capturing evolving interactions between individuals (such as interactions between students in a high school) and biological networks capturing interactions between biomolecules in the cell at different ages. All dynamic network data used in this study have labeled nodes, i.e., metadata (this is exactly why we have chosen these networks—they are among very few publically available datasets that are both dynamic and have metadata). For example, for the social data, a node can be labeled as a student or a teacher, while for the biological data, a node can be labeled as aging-related or non-aging-related. By having the label for each node, we know the ground truth partition, meaning that nodes with the same label should be in the same cluster. Thus, we evaluate how well the partition generated by each method corresponds to the ground truth partition. For this evaluation, we use the following established partition quality measures: precision, recall, F-score, and adjusted for chance mutual information [8, 28]. We evaluate the statistical significance of each partition quality score.

We find that across all partition quality measures and all considered social datasets, when compared to the existing methods, C-ST is the best method 55.5% of the time, C-D is the best method 77.7% of the time, and C-C is the best method 89% of the time. Across all partition quality measures and all considered biological datasets, when compared to the existing methods, C-ST is the best method 83% of the time, C-D is the best method 75% of the time, and C-C is the best method 41.6% of the time. Importantly, on both the social and biological datasets, no matter which of C-ST, C-D, and C-C is used, ClueNet is overall superior to the existing approaches. Of the three ClueNet versions, C-D is more accurate than C-ST on the social datasets, as expected, but C-ST is surprisingly better than C-D on the biological datasets. In terms of C-D versus C-C, C-C is faster than C-D on all datasets, as expected. Yet, in terms of accuracy, C-C is surprisingly also better than C-D on the social datasets, while C-D is superior on the biological datasets, as expected.

Our results validate our two hypotheses that considering topological similarity as a notion of topological relatedness of nodes instead of interconnectivity denseness, as well as that explicitly capturing inter-snapshot information before performing any clustering rather than doing so implicitly after the initial clustering is done, can yield better partitions. Regarding the former, we show an example where combining topological similarity of ClueNet and interconnectivity denseness of an existing method improves the output of each of the two individual methods. Exploring whether this observation holds systematically is certainly of future interest, but it is out of the scope of the current study.

## Methods

First, we introduce definitions that will be used throughout the paper. Second, we describe our new ClueNet approach. Third, we give an overview of the existing methods that we compare against, and we also describe how dynamic graphlets can be applied on top of some of the existing methods. Fourth, we describe the temporal data that we evaluate the methods on. Fifth, we discuss parameter values that we use to construct dynamic networks from the temporal data. Sixth, we describe partition quality measures that we rely on. Seven, we discuss parameter values that we use to cluster the dynamic networks.

### Definitions

**Dynamic network**. As illustrated in Fig 1, we need to distinguish between: 1) a raw temporal network dataset that contains the following information about each event: when the given event occurs, between which elements (i.e., nodes) it occurs, and how long it lasts; and 2) the snapshot-based representation of the raw dataset (defined below). Sometimes, we are explicitly provided with the snapshot-based representation of a raw temporal dataset, in which case each considered method can be directly run on such data. Other times, we are provided with a raw temporal dataset, in which case the dataset needs to be converted into the snapshot-based representation prior to running the given method. This is typically done as follows. The entire time interval of the raw temporal data is split into time windows of size *t*_*w*_. For each window, a static network snapshot is generated such that an edge between nodes *u* and *v* will exist if and only if there are at least *w* events between *u* and *v* during the given window (Fig 1). The series of snapshots generated from this process form the snapshot-based representation of the dynamic network. More formally, a raw temporal network dataset *D*(*V*, *E*), where *V* is the set of nodes and *E* is the set of events, can be represented as a sequence of *m* network snapshots {*G*_0_, *G*_1_,…, *G*_*m*_}, where each snapshot *G*_*i*_ = (*V*_*i*_, *E*_*i*_) is a static graph capturing network structure during time interval *i* (as described above), and *V*_*i*_ ⊆ *V* and *E*_*i*_ ⊆ *E* (where *E*_*i*_ is restricted to nodes in *V*′).

**Static and dynamic graphlets**. Static graphlets are small connected non-isomorphic induced subgraphs of a static network that can be used to capture the extended neighborhood of a node. In other words, a static graphlet is an equivalence class of isomorphic connected subgraphs. Dynamic graphlets [5], which are an extension of static graphlets [3] for dynamic networks, are dynamic subgraphs that can be used to capture how the extended neighborhood of a node evolves across snapshots of a dynamic network. Intuitively, dynamic graphlets add relative temporal order of events on top of static graphlets (for a formal definition, see S1 Section).

For the purpose of this study, we analyze all static graphlets with up to four nodes and thus up to six edges, and we analyze dynamic graphlets with up to four nodes and up to six events, as this dynamic graphlet size was suggested as “optimal” in the original paper [5].

**Static-temporal graphlet degree vector (ST-GDV) of a node**. Given all static graphlets on up to *n* nodes, in each snapshot, we can count how many times a node touches each of the static graphlets, and in particular how many times a node touches each of the graphlets at each of their automorphism orbits (or node symmetry groups) [3]. Our considered static graphlets on up to four nodes have 15 orbits. So, for each node in a given snapshot of the dynamic network, we count the number of times the given node touches each of the 15 orbits. We do this for each of the *m* snapshots. Then, for a given node, we concatenate its *m* 15-dimensional counts. The resulting *m* × 15-dimensional vector is ST-GDV of the node, which captures the extended neighborhood of the node in the dynamic network in a static-temporal fashion (i.e., by counting static graphlets in individual snapshots and then integrating the counts over the different snapshots).

**Dynamic graphlet degree vector (D-GDV) of a node**. Given all dynamic graphlets on up to *n* nodes and up to *j* events, we can count how many times a node touches each of the dynamic graphlets at each of its orbits [5]. Our considered dynamic graphlets on up to four nodes and up to six events have 3,727 orbits. So, for each node in a dynamic network, we count the number of times the given node touches each of the 3,727 orbits. The resulting 3,727-dimensional vector is D-GDV of the node, which captures the evolutionary behavior of the extended neighborhood of the node in the dynamic network.

**Constrained dynamic graphlet degree vector (C-GDV) of a node**. Counting of dynamic graphlets in a network can be computationally expensive when there is a large number of occurrences of a given dynamic graphlet. Since multiple occurrences of a given dynamic graphlet can often be artifacts of consecutive snapshots sharing the same network structure, the notion of constrained counting of dynamic graphlets in a network was introduced. This is a modification of the counting process that does not count “redundant” dynamic graphlets, i.e., dynamic graphlets that result from consecutive snapshots sharing the same network structure. That is, this modification, which we denote by C-GDV, still counts the number of times the given node touches each of the 3,727 orbits, but its counts will be typically smaller than those of the node’s D-GDV. Subsequently, it takes less time to compute the node’s C-GDV than it takes to compute its D-GDV. More details about this modified counting procedure can be found in [5].

**Partition**. A partition *P* is the division of nodes in a network into clusters that satisfies the following three conditions: each cluster is non-empty, no two clusters overlap, and the union of all clusters is the set of all nodes in the network.

### ClueNet

ClueNet produces a single partition of the nodes of a dynamic network. When generating a partition, ClueNet aims to group together (i.e., cluster) nodes that have similar evolving (extended) network neighborhoods, as reflected by the similarity of the nodes’ ST-GDVs, D-GDVs, or C-GDVs. Below, we explain ClueNet on the example of using D-GDVs, but the process is identical if ST-GDVs or C-GDVs are used instead.

In order to generate a partition, ClueNet must first compute the D-GDV of each node. However, it might be undesirable for ClueNet to simply use the raw D-GDV counts, due to the high dimensionality of each D-GDV. The dimensionality of a given D-GDV could be an issue because there will most likely be many orbits with the value of 0. Thus, comparing D-GDVs of two nodes that both have many orbits with the value of 0 could artificially make the nodes appear more similar then they actually are, because the total similarity could be dominated by the 0-value orbits. In order to reduce the impact of this issue as well as to select the most important (discriminative) of all non-0 orbits, we apply principal component analysis (PCA) to the D-GDVs, which reduces the dimensionality of the vectors. For this study, we use as few PCA components that contain at least 90% of the variance. We note that while ST-GDVs do not have as high dimensionality as D-GDVs or C-GDVs, we still apply the above process to ST-GDVs as well, for a fair comparison of the three ClueNet versions.

Given the selected PCA components for each node in the dynamic network, ClueNet computes distances (or equivalently, similarities) between each pair of nodes in the network. Intuitively, the more similar the dynamic network neighborhoods (i.e., PCA-reduced D-GDVs) of two nodes, the lower the distance between the nodes should be. As a proof of concept, we evaluate two distance/similarity measures: Euclidean distance and cosine similarity. We use these measures due to their popularity, and due to their different approaches to measuring distance between vectors (i.e., linear distance versus difference in orientation). For each dataset, we report results for the measure that generates the highest partition quality (Section Measuring partition quality). The pairwise node distances are stored into the D-GDV similarity matrix.

Finally, ClueNet plugs the D-GDV similarity matrix into the *k*-medoids algorithm (see S2 Section for details). The number of clusters *k* is a parameter of this algorithm. Later on in the paper, we describe our procedure of choosing this parameter value. The output of the *k*-medoids clustering algorithm is a single partition of the network, which we treat as the output of ClueNet.

Given that we utilize three different methods of graphlet counting, namely ST-GDVs, D-GDVs, and C-GDVs, we have three versions of ClueNet, namely C-ST, C-D, and C-C, respectively.

The code for ClueNet is available at http://www.nd.edu/~cone/cluenet.

### Existing methods

Five of the existing DNC methods that we consider were originally proposed in the static network clustering setting and were then extended to the dynamic setting. For the description of how each method clusters a static network, see S3 Section. All of the (static versions of the) methods work on a weighted static network, which allows them to be adapted to solve the DNC problem, as follows. Given a dynamic network with *p* snapshots, each with the same *n* nodes, the given clustering algorithm *A* (out of the above static methods) is used to generate *p* partitions, one partition per snapshot. Then, an *n* × *n* consensus matrix *D* is generated, where each matrix element *D*_*ij*_ indicates the number of partitions (out of the *p* partitions) in which nodes *i* and *j* are clustered together. Matrix *D* is essentially a weighted matrix that can be transformed into a weighted static network, in which nodes are the same nodes as in the original dynamic network, and edges are weighted according to matrix *D*. That is, a *D*_*ij*_ value of 0 in matrix *D* indicates that nodes *i* and *j* are not connected in the weighted static network, and a *D*_*ij*_ value greater than 0 indicates that nodes *i* and *j* are connected in the static network with the weight *D*_*ij*_. Algorithm *A* is then applied on the resulting weighted static network *p* times. If all resulting *p* partitions are equal, the algorithm halts and outputs any one of the *p* partitions as the consensus partition. Otherwise, matrix *D* is regenerated using the newly-generated *p* partitions and the process is repeated until all *p* partitions are equal. For full details of how the static clustering methods were transformed to solve the DNC problem, see the original paper [10].

The sixth of the existing DNC methods that we consider was proposed directly in the dynamic setting (S4 Section).

### Integrating dynamic graphlets into existing DNC methods

The existing DNC methods define a cluster as a densely interconnected network region. As such, they typically cannot cluster together nodes that are far apart in the network, despite the fact that nodes with the same metadata (i.e., with the same labels), which should thus be clustered together, can be spread throughout the network, without necessarily being close to each other [4]. Instead, our ClueNet approach clusters nodes with similar extended network neighborhoods. Nodes that are densely interconnected with each other may have similar neighborhoods simply because they are in the same neighborhood. Thus, our approach could capture the same “signal” as the existing denseness-based approaches. At the same time, it can capture nodes that have similar neighborhoods but are not close in the network. Yet, our approach does not guarantee to be able to capture densely interconnected nodes. So, to benefit from both worlds, i.e., topological similarity and dense interconnectedness, we perform a case study (for a network whose metadata supports the idea of dense interconnectedness) of whether combining topological similarity of ClueNet and interconnectivity denseness of an existing method improves the output of each of the two individual methods. To achieve this, instead of using the *n* × *n* consensus matrix within an existing method (Section Existing methods and S3 Section), we use ClueNet’s topological (D-GDV) similarity matrix. Then, all steps of the existing method are performed on the D-GDV similarity matrix.

### Data

We evaluate all methods on four dynamic networks: three social networks and one biological network.

The three temporal raw social datasets that are used to construct the three corresponding dynamic networks are as follows. The *Enron* dataset [29] is based on email communications of 182 employees between the years 2000 and 2002, with each employee having one of seven labels representing roles at the company (for the list of labels and the number of employees having the given label, see S1 Table). In the corresponding dynamic network, nodes are employees and events are email communications between the employees. The *hospital* dataset [30] corresponds to contacts between 75 patients and healthcare workers over four days at a hospital in Lyon, France, with each person having one of four labels representing positions at the hospital (for the list of labels and the number of people having the given label, see S2 Table). In the corresponding dynamic network, nodes are patients and events are contacts between the patients. The *high school* dataset [31] corresponds to contacts between 327 students in a high school in Marseilles, France over five days, with each student having one of nine labels representing classes (for the list of labels and the number of students having the given label, see S3 Table). In the corresponding dynamic network, nodes are students and events are contacts between the students. The Enron dataset is available for download at http://cis.jhu.edu/~parky/Enron/enron.html and the remaining two social network datasets are available for download at http://www.sociopatterns.org/datasets/. Network construction parameters that we use to transform each of the raw temporal social datasets into the corresponding dynamic network are discussed below.

The biological dataset that we use is already provided in the form of a snapshot-based dynamic network. The dynamic network captures age-specific protein-protein interaction (PPI) network snapshots for 37 ages between 20 and 99 [32]. That is, in the network, nodes are proteins, and events are PPIs between them. There are 6,465 nodes in the network. Each age-specific snapshot was constructed by taking an induced subgraph of the currently static human PPI network on proteins that are significantly expressed (i.e., active) at the given age according to an aging-related gene expression dataset [33]; for details, see the original study [32]. In each snapshot, we consider a node to be either aging-related or currently not implicated in aging, based on each of the following four ground truth aging-related datasets: two brain aging-related datasets that were obtained via gene expression analyses (denoted as BE2004 and BE2008 [32]), a dataset related to Alzheimer’s disease that was obtained via gene expression analyses (denoted as AD) [34], and a dataset consisting of human genes that have aging-related orthologs in model species, which was obtained via sequence-based analyses (denoted as SequenceAge) [35]. BE2004, BE2008, AD, and SequenceAge contain 310, 3,859, 1,349, and 189 genes, respectively, that are also found within our biological network dataset [33]. If a gene is in the given ground truth aging-related dataset, then it is considered to be aging-related. Otherwise, the gene is considered as currently not implicated in aging, which for simplicity we refer to as non-aging-related (keeping in mind that some of these genes might be implicated in aging in the future).

### Dynamic network construction parameters

While the biological dataset is already given to us as a snapshot-based dynamic network, the three social datasets are not. For each of these three datasets, recall from Section Definitions that constructing a snapshot-based dynamic network requires choosing values of two parameters: time window size *t*_*w*_ and number of events *w*. For each of the three datasets, we evaluate each DNC method on multiple combinations of *t*_*w*_ and *w* parameters. Then, we use the parameter combination that yields the highest-quality partition, i.e., that results in the highest geometric mean of precision, recall, and AMI partition quality measures (these measures are described below). This way, we give the best-case advantage to each method on each dataset. For the Enron dataset, we explore the established parameter values [5]. Namely, we test all possible combinations of the following *t*_*w*_ and *w* values:

*t*_*w*_ = {1 week, 2 weeks, 1 month, 2 months, 3 months} and *w* = {1, 2, 4, 8, 16}. For the hospital dataset, we test all possible combinations of the following *t*_*w*_ and *w* values:
*t*_*w*_ = {50, 100, 200, 300, 500} minutes and *w* = {1, 2, 4, 8, 16, 32}. For the high school dataset, we test all possible combinations of the following *t*_*w*_ and *w* values:
*t*_*w*_ = {25, 50, 100, 200, 300, 500} minutes and *w* = {1, 2, 4, 8, 16, 32, 64}. Note that the *t*_*w*_ and *w* values that we evaluate differ between the three datasets. This is because the datasets have different time scales.

The best parameter values for each DNC method on each dataset, which are used when reporting the methods’ results, can be found in S4 Table.

### Measuring partition quality

Given a partition of a dynamic network obtained by a given DNC method, and given the ground truth partition obtained with respect to node labels (so that nodes with the same label are in the same cluster), we evaluate the quality of the given method’s partition by measuring precision, recall, F-score, and the adjusted for chance mutual information (AMI).

Precision is computed as $\frac{\#\;\text{of}\;\text{true}\;\text{positives}}{\#\;\text{of}\;\text{true}\;\text{positives}+\;\#\;\text{of}\;\text{false}\;\text{positives}}$, where a true positive is a pair of nodes that have the same label and are also in the same cluster, and a false positive is a pair of nodes that have different labels but are in the same cluster. Recall is computed as $\frac{\#\;\text{of}\;\text{true}\;\text{positives}}{\#\;\text{of}\;\text{true}\;\text{positives}+\;\#\;\text{of}\;\text{false}\;\text{negatives}}$, where a true positive is defined above, and a false negative is a pair of nodes that have the same label but are in different clusters. F-score is the harmonic mean of precision of recall. AMI [28] determines how similar a given method’s partition and a ground truth partition are from an information-theoretic perspective. Given the two partitions, AMI quantifies how much knowing one partition reduces the uncertainty about knowing the other. For all measures, the higher the score, the better the partition.

A method must be able to generate a partition of statistically significantly high quality. That is, a high partition quality score is not necessarily sufficient. For example, by producing a partition with a single cluster containing all nodes in the network, the given method’s partition quality score in terms of recall would have the maximum possible value of 1. However, such a high score is meaningless, since any random partition with the same number of clusters (in our illustration, one cluster) and with clusters of the same size (in our illustration, all the nodes) would also have the maximum possible recall value of 1. So, we evaluate the statistical significance of each partition quality score, and we do so by relying on as strict null model as possible, to ensure that it is not the number of clusters or the cluster sizes that yield the given partition quality score. Specifically, for each partition generated by a DNC method, we generate a random partition that has the same number of clusters as well as clusters of the same sizes as the given method’s partition, but node memberships in the clusters are randomized compared to the given method’s partition. For each considered partition, we perform this randomization process 5,000 times. Then, we compute the *p*-value of the given partition quality score observed in the actual method’s partition as the percentage of the 5,000 random runs that have the same or higher partition quality score. We say that one method is superior to another method if the former has a lower *p*-value than the latter. Otherwise, if the compared methods have the same *p*-value, we compare the methods’ raw partition quality scores. We use the *p*-value of 0.05 (5%) to determine whether the given partition quality score is significant or not.

### Choosing the number of clusters for the *k*-medoids algorithm with ClueNet

The *k*-medoids algorithm allows the user to select the desired number of clusters *k*. We determine empirically in a systematic way the “optimal” value of *k* for each network. For the given network, we look at all possible values of *k* (between 1 and the number of nodes in the network) for which the *k*-medoids algorithm takes no longer than one hour to run on a Linux machine with 64 cores (AMD Opteron^™^ Processor 6378) and 512 GB of RAM. We limit the running time intentionally, because otherwise, it is possible that the algorithm may be looping through the same centroids. For each of the resulting values of *k*, due to the *k*-medoids algorithm being heavily reliant on the initial centroids selected, we run the algorithm 10 times, and we choose the partition that occurs the most frequently among the 10 partitions. Given one resulting partition for each value of *k*, we choose the value of *k* that maximizes the geometric mean of the corresponding partition’s precision, recall, and AMI scores (we leave out its F-score, since F-score is somewhat redundant to precision and recall).

## Results and discussion

First, we look at three dynamic social networks (Enron, high school, and hospital; Section Data) and one dynamic biological network (related to aging; Section Data) and their corresponding node metadata-based ground truth partitions (one partition for each of the three social networks and four partitions for the biological network; Section Data), and we examine properties of the seven ground truth partitions with respect to topological similarity (as captured by our D-GDV-based node similarity measure; Section Definitions) versus dense interconnectedness (as captured by the existing modularity measure). If the ground truth partitions reflect high topological similarity, this would motivate the need for our proposed topological similarity-based ClueNet approach. Also, if the partitions reflect low modularity, this would question the typical assumption of the existing approaches. Second, we apply ClueNet (each of its three versions: C-ST, C-D, and C-C; Section ClueNet) and the existing DNC methods (Louvain, Infomap, Hierarchical Infomap, label propagation, simulated annealing, and Multistep; Section Existing methods and S3 Section) to each of the four networks, and we contrast the different methods with respect to four measures of partition quality (precision, recall, F-score, and AMI; Section Measuring partition quality) that evaluate how well the methods capture the ground truth partitions. Third, we mix-and-match the ideas of topological similarity-based clustering and denseness-based clustering (Section Integrating dynamic graphlets into existing DNC methods) to see if this improves compared to each of the two ideas individually. Fourth, we discuss running times of the methods.

### What data tell us about the importance of topological similarity versus dense interconnectedness

Here, we provide evidence that the real-world data that we study question the dense interconnectedness assumption of the existing DNC methods and also motivate the need for our topological similarity assumption.

**Fig 5:**
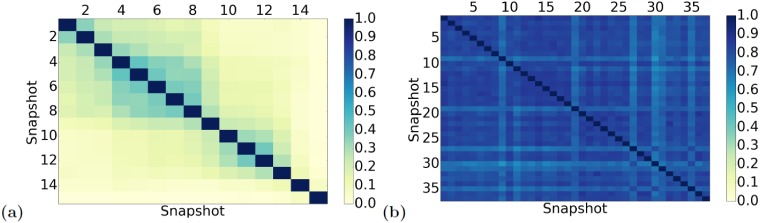
Pairwise edge overlaps between the snapshots of (a) social Enron and (b) biological aging-related dynamic networks. The darker the color, the higher the edge overlap between the given snapshots. For the Enron data, the following network construction parameter values are used: *t*_*w*_ = 2 months and *w* = 2, but the results are similar for the other tested parameter values. Equivalent results for the other two social networks (hospital and high school), which are similar to the Enron results, are shown in S1 Fig.

First, the existing DNC methods assume that nodes should be in the same cluster if they are densely interconnected in many of the snapshots of the given dynamic network. However, if there is very little edge overlap between the different snapshots, it could be difficult for same nodes to be densely interconnected in many of the snapshots. Interestingly, low across-snapshot edge overlap is exactly what we see in all of our analyzed social networks (Fig 5(a) and S1 Fig), though not in the analyzed biological network (Fig 5(b)).

**Fig 6:**
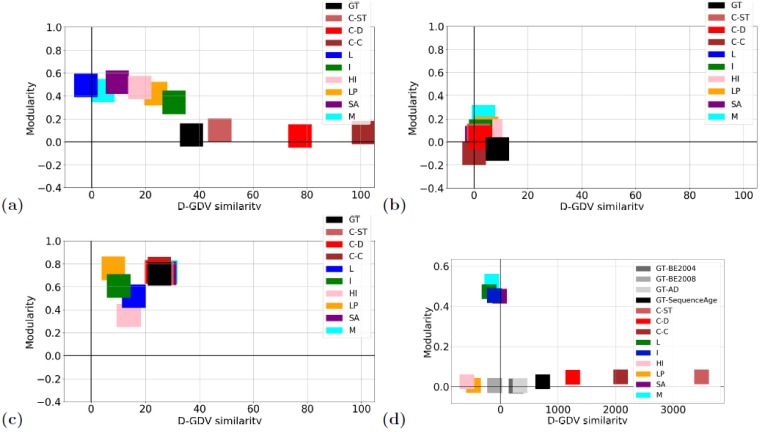
The fit of each method’s partition to the ground truth partition(s). The fit of each method’s partition (the methods are: ClueNet (its three versions: C-ST, C-D, and C-C), Louvain (L), Infomap (I), Hierarchical Infomap (HI), label propagation (LP), simulated annealing (SA), and Multistep (M)) to the ground truth (GT) partition(s), for **(a)** social Enron, **(b)** social hospital, **(c)** social high school, and **(d)** biological aging-related dynamic networks, with respect to topological (D-GDV) similarity versus interaction denseness (modularity). In the given panel, a method is good if its partition is in the same quadrant as the ground truth partition and if the two partitions both show high or low D-GDV similarity and modularity scores. In panel (a), only the three ClueNet versions match both high D-GDV similarity and low (close to 0 but positive) modularity scores of the ground truth partition. In panel (b), all three versions of ClueNet are closer to the ground truth partition than the existing methods. Note that in panel (b), the Louvain method is missing, because it did not produce any output for this network. In panel (c), all methods mimic well both high D-GDV similarity and high (positive) modularity scores, but the three ClueNet versions are the closest to the ground truth partition, along with simulated annealing and Multistep. Note that all five of these methods produce the exact same partition. So, their visualizations have been slightly manipulated by moving some of the methods’ results just a bit up/down or left/right, in order to make all five methods visible. In panel (d), there are four ground truth partitions, depending on which aging-related ground truth data is considered (BE2004, BE2008, AD, or SequenceAge; Section Data). For three of the four ground truth partitions, only the three versions of ClueNet match both high (positive) D-GDV similarity and low (close to 0 but positive) modularity scores.

Second, if the dense interconnectedness assumption of the existing DNC methods is valid, then the ground truth partitions of the analyzed networks should show high modularity. However, for two of the three social ground truth partitions (corresponding to the Enron and hospital networks) as well as for all four of the biological ground truth partitions, modularity is random-like (negative or close to 0) (see only the “GT” squares in Fig 6(a), 6(b) and 6(d)). Only one of the social ground truth partitions (corresponding to the high school network) has high modularity (see only the “GT” square in Fig 6(c)). Thus, the majority (6/7 = 86%) of the analyzed ground truth partitions do *not* support the existing idea of dense interconnectedness.

On the other hand, all three social ground truth partitions and three out of the four biological ground truth partitions show statistically significantly high D-GDV similarity (Fig 6). That is, 6/7 = 86% of the analyzed ground truth partitions *do* support our proposed idea of topological similarity. Here, by statistically significantly high D-GDV similarity, we mean that node pairs that are in the same cluster of the given partition have statistically significantly higher D-GDV similarity than node pairs that are in different clusters of the same partition, i.e., that according to the Wilcoxon rank sum test, the raw score of the test is positive and the *p*-value of the score is below 0.05).

### Method comparison in terms of accuracy

In terms of accuracy, for each analyzed network and its corresponding ground truth partition(s), we compare the three versions ClueNet to the existing methods in the following two ways, by measuring: 1) how well each method’s partition fits the ground truth partition, in terms of modularity as well as D-GDV similarity, and 2) which method yields the highest-quality partition in terms of precision, recall, F-score, and AMI.

For the latter, for each method, each network/ground truth partition(s), and each partition quality measure, we evaluate whether the given partition quality score is statistically significantly high (Section Measuring partition quality). Then, we consider method *a* to be superior to method *b* if the *p*-value of method *a* is lower than the corresponding *p*-value of method *b*. If multiple methods have the same *p*-value, we then compare the methods’ raw partition quality scores. Finally, across all evaluation tests, i.e., all analyzed networks/ground truth partitions (three partitions for the three social networks, and four partitions for the biological network) and all partition quality measures (actually, across precision, recall and AMI, leaving out F-score that incorporates precision and recall and is thus redundant to them), we compute for each method the percentage of all evaluation tests in which the given method is the best of all methods (rank 1), the second best of all methods (rank 2), the third best of all methods (rank 3), and so on. We deal with ties in ranks as follows: in the case that multiple methods both have rank *k*, the next highest-ranking method(s) will be given rank *k* + 1.

#### Social datasets

With respect to the fit of each method’s partition to the corresponding ground truth partition(s) in terms of modularity and D-GDV similarity, we observe that ClueNet’s partitions overall match the ground truth partitions the best (Fig 6(a)–6(c)). While some of the existing DNC methods’ partitions also match well the ground truth partitions of the hospital and high school networks (but not the ground truth partition of the Enron network), each of the three ClueNet versions fits these ground truth partitions better than the existing methods.

**Fig 7:**
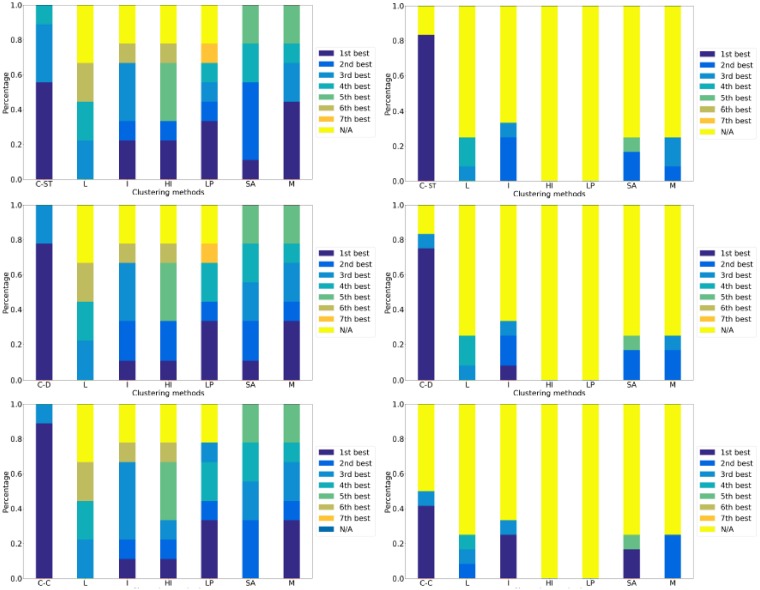
The ranking of all DNC methods used in this study. The ranking of the methods (ClueNet (its three versions: C-ST, C-D, and C-C), Louvain (L), Infomap (I), Hierarchical Infomap (HI), label propagation (LP), simulated annealing (SA), and Multistep (M)) over all considered social datasets (i.e., the three ground truth partitions corresponding to the three social dynamic networks; the first column) and **(b)** biological datasets (i.e., the four ground truth partitions corresponding to the biological aging-related dynamic network; the second column) with respect to all of precision, recall, and AMI (F-score is excluded here because it is redundant to precision and recall). Each row corresponds to one of the three versions of ClueNet that is compared to the existing methods: C-ST (top), C-D (middle), and C-C (bottom). The ranking is expressed as a percentage of all cases (across all ground truth partitions and all three partition quality measures) in which the given method yields the *k*^*th*^ best score across all methods. We rank the methods based on their *p*-values (i.e., the smaller the *p*-value, the better the method); in case of ties, we compare the methods based on their raw partition quality scores. The ‘N/A’ rank signifies that the given method did not produce a statistically significant partition under the given partition quality score.

With respect to the ranking of the different methods, across all three social network datasets (i.e., the three networks and their three ground truth partitions) and the three partition quality measures (precision, recall, and AMI), *each* version of ClueNet (C-ST, C-D, and C-C) is better than *all* existing methods (Fig 7). When compared to the existing methods only, C-ST is the best of all methods 55.5% of the time, C-D is the best of all methods 77.7% of the time, and C-C is the best of all methods 89% of the time. Importantly, all three versions of ClueNet always result in statistically significant partitions. Only two of the existing methods (simulated annealing and MultiStep) also show this trend.

When comparing the performance of the three versions of ClueNet, C-C is the best, as it is dominant to the existing methods in more cases than C-D and C-ST (Fig 7 and S2 Fig). Of the remaining two versions, C-D is superior to C-ST. Note that whenever C-ST has rank 1, it is not actually superior to C-D and C-ST. Instead, it is tied to them. Hence, (constrained) dynamic graphlets outperform static graphlets, as expected.

Note that for the high school and hospital datasets, five out of the six ClueNet’s results (corresponding to the two datasets times the three ClueNet versions) come from applying dynamic graphlets on top of one of the existing dense interconnectedness-based DNC methods (namely simulated annealing, see Section Integrating dynamic graphlets into existing DNC methods for details). For the remaining ClueNet’s result on these datasets, as well as for all three results (corresponding to the three ClueNet versions) for the Enron dataset, the default *k*-medoids version of ClueNet is used, as described in Section ClueNet and S2 Section. See S4 Table for more details.

**Fig 8:**
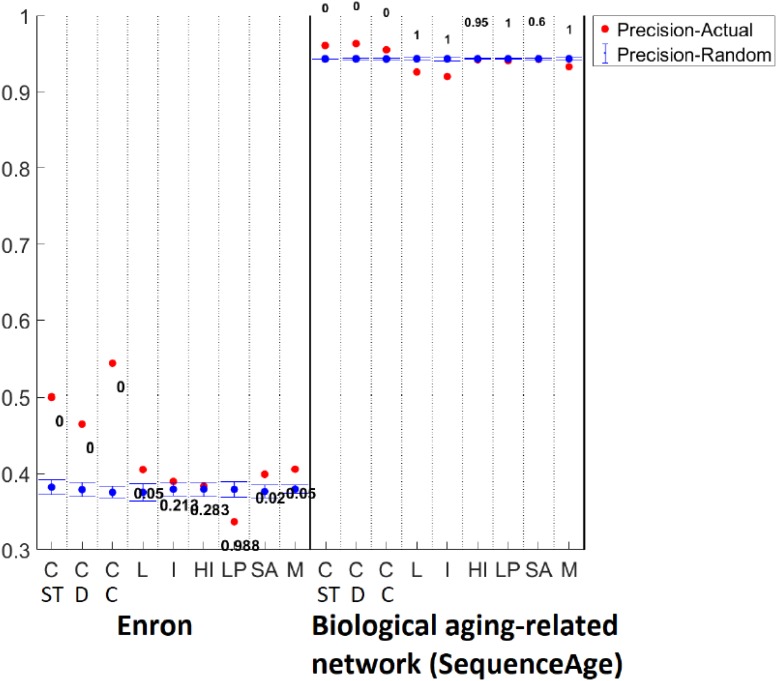
Detailed method comparison. Detailed method comparison results for the social Enron (left) and biological aging-related (right) dynamic networks, quantifying the fit of each method (ClueNet (C-ST,C-D,C-C), Louvain (L), Infomap (I), Hierarchical Infomap (HI), label propagation (LP), simulated annealing (SA), and Multistep (M)) to the corresponding ground truth partition in terms of precision. There is one ground truth partition for the Enron network (results shown in the figure). There are four ground truth partitions for the aging-related networks, depending on which aging-related ground truth data is considered (BE2004, BE2008, AD, or SequenceAge; Section Data). Results are shown in this figure for the SequenceAge-based ground truth partition. For each dataset, for each method, we compare the precision score of the partition produced by the given method (red) to the average precision score of its random counterparts (blue) and show the resulting *p*-value (see Section Measuring partition quality for details). These are representative results for one network/ground truth partition from each of the social and biological domains and one measure of partition quality. Equivalent results for the other three biological aging-related ground truth partitions, for the other two social dynamic networks (hospital and high school), and for the other three partition quality measures (recall, F-score, and AMI) are shown in, S3, S4, S5 and S6 Figs.

Detailed versions of the above results that show all raw partition quality scores as well as their *p*-values, which support the above finding that ClueNet is overall superior, are shown in Fig 8, S3, S4, S5 and S6 Figs.

#### Biological datasets

With respect to the fit of each method’s partition to the corresponding ground truth partition(s) in terms of modularity and D-GDV similarity, all versions of ClueNet again overall match the best three of the four aging-related ground truth partitions (Fig 6(d)). While Hierarchical Infomap and label propagation also fit one of the four partitions, the remaining DNC methods show no fit whatsoever to any of the partitions.

With respect to the ranking of the different methods, across all four ground truth datasets (i.e., four ground truth partitions for the biological network) and the three partition quality measures (precision, recall, and AMI), again, *each* version of ClueNet (C-ST, C-D, and C-C) is better than *all* existing methods (Fig 7). When compared to the existing methods only, C-ST is the best of all methods 83% of the time, C-D is the best of all methods 75% of the time, and C-C is the best of all methods 41.6% of the time. All three versions of ClueNet always result in most of statistically significant partitions. Interestingly, some of the existing methods completely fail on the biological datasets, as they never produce a statistically significant partition.

When comparing the performance of the three versions of ClueNet, C-ST is surprisingly superior, followed by C-D and C-C, respectively (Fig 7 and S2 Fig). Hence, for the biological datasets, static graphlets are superior to (constrained) dynamic graphlets. This is the opposite from what we have seen for the social datasets. We hypothesize that this might be due to the properties of the considered social and biological networks: while for each social network, its snapshots are quite dissimilar, with low edge overlaps, for the biological network, its snapshots are very similar (i.e., stable), with high edge overlaps (Fig 5). Thus, it could be that dynamic graphlets are not as successful in capturing stability in dynamic networks as static graphlets are, but they could be extended in the future to account for snapshot stability. This is just a potential explanation, which would need to be examined systematically in carefully controlled experiments. This is out of the scope of this study and is the subject of our future work.

Detailed versions of the above results that show all raw partition quality scores as well as their *p*-values, which support the above finding that ClueNet is overall superior, are shown in are shown in Fig 8, S3, S4, S5 and S6 Figs.

### Combining ideas of topological similarity and dense interconnectedness

Recall from Section Integrating dynamic graphlets into existing DNC methods that we are able to use the notion of dynamic graphlets, i.e., D-GDV similarity, as input into an existing DNC method. Thus, we are able to evaluate whether using topological D-GDV similarity on top of an existing DNC method can improve results of the existing DNC method in question. We perform this analysis on all existing DNC methods used in this study that allow us to input a custom (in our case, D-GDV-based similarity) similarity matrix. While for most of the methods, using D-GDV similarity results in more or less comparable results, there is a significant improvement for one of the methods, namely simulated annealing, on the high school dataset. That is, by using the D-GDV similarity matrix as input into simulated annealing, we are not only able to improve upon both ClueNet and simulated annealing (Table 1), but this combination of D-GDV similarity and simulated annealing becomes one of the best performing methods (S3, S4, S5 and S6 Figs). For example, this combination is one of the methods that overlaps the most with the ground truth high school partition with respect to AMI (S7 Fig). The results illustrate the potential of combining the notions of topological similarity and dense interconnectedness, especially for networks that do incorporate the dense interconnectedness assumption, such as the high school dataset. Similar results hold for C-ST and C-C instead of C-D (S5 Table) on this dataset.

**Table 1:** Results when ClueNet’s dynamic graphlet-based topological similarities are used on top of the existing denseness-based simulated annealing method.

	Precision	Recall	F-Score	AMI
**Original simulated annealing**	96.68%	93.89%	89.83%	0.9372
**ClueNet (C-D), i.e., dynamic graphlets**	14.73%	37.27%	21.11%	0.1467
**Simulated annealing with dynamic graphlets**	99.44%	99.39%	99.41%	0.993

Partition quality results of the original denseness-based simulated annealing, ClueNet (C-D), and when combining ClueNet’s dynamic graphlet-based topological similarities with simulated annealing, for the high school dataset.

Note that low accuracy in Table 1 of C-D, i.e., of dynamic graphlets alone, for the high school dataset could be explained as follows. Recall from Fig 6 that this is the only considered dataset whose ground truth partition has high modularity (see the “GT” squares in Fig 6). Dynamic graphlets that C-D is based on capture information about the (extended) network neighborhood of a node and thus about topological similarity between nodes, independent of whether the nodes are densely interconnected. Hence, dynamic graphlets do not necessarily capture well the notion of dense interconnectedness (i.e. modularity). In other words, dynamic graphlets may have difficulty in generating a high-quality partition with respect to modularity, in which case they would not match well a ground truth partition that has high modularity, such as that for the high school dataset.

### Method comparison in terms of running time

Here, we compare ClueNet to the other DNC methods in terms of running time. All methods are run on a Linux machine with 64 cores (AMD Opteron^™^ Processor 6378) and 512 GB of RAM. Each method is run on a single core. We account for the entire running time of each DNC method, which includes the computation of graphlets (if applicable), the computation of node similarities, and the creation of the partition.

**Fig 9:**
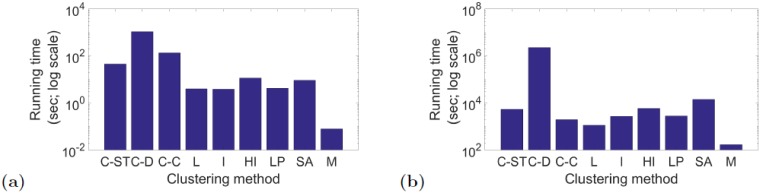
Running time comparison. Running time comparison of the different methods (ClueNet (its three versions: C-ST, C-D, and C-C), Louvain (L), Infomap (I), Hierarchical Infomap (HI), label propagation (LP), simulated annealing (SA), and Multistep (M)) for the **(a)** social Enron and **(b)** biological aging-related dynamic networks. On the *y*-axis, log base 10 is used. Equivalent results for the other two social networks (hospital and high school), which are similar to the Enron results, are shown in S8 Fig.

With respect to running times, Multistep is generally the fastest out of all methods, while ClueNet typically has the highest running time (Fig 9 and S8 Fig). This is because ClueNet’s running time is dominated by the process of graphlet counting. For example, for C-D, the percentage of the entire running time that is spent on counting the dynamic graphlets for the Enron, hospital, high school, and biological networks is 97.3%, 18.3%, 51.5%, and 99.9%, respectively. However, once dynamic graphlets are counted, they do not need to be counted again. Also, C-C, the best version of ClueNet for the social networks, offers speed-up compared to C-D. Similarly, C-ST, the best version of ClueNet for the biological network (the largest of all analyzed networks, with ∼6, 500 nodes), is faster than C-D, and its running time is actually comparable to running times of most of the existing methods.

Note that for our considered social data, Multistep can be considered as a viable alternative to our ClueNet approach because Multistep is faster than ClueNet as well as the second best method overall and the best method in some evaluation tests. However, for our considered biological data, Multistep is only the fourth best method overall and it is not the best method in any evaluation test. So, Multistep’s performance is data-dependent. On the other hand, ClueNet performs consistently well on both the social and biological data. Further, on the biological data, none of the existing methods that are faster than ClueNet can compare to ClueNet when it comes to accuracy. Thus, even with the current (computationally intensive) implementation of C-ST, C-D and C-C, their higher running times compared to the existing approaches are justified by their higher accuracy, at least in networks where graphlet counting is feasible.

Further, there exist methods that speed up static graphlet counting in even larger networks [36, 37], even with millions of nodes [38]. So, these methods can be used to count graphlets within the C-ST version of our ClueNet approach. Similar speed-up extensions could be pursued in the future for dynamic graphlet counting as well, to improve scalability of the C-D version of our ClueNet approach. While this is certainly of our interest, such an extension is out of the scope of this study.

## Conclusion

We introduce ClueNet, a new DNC approach that overcomes the two key advantages of the existing approaches: ClueNet clusters nodes based on regular equivalence (i.e., topological similarity), while the existing approaches cluster based on structural equivalence (i.e., dense interconnectedness), and also, ClueNet (its C-D and C-C versions) captures inter-snapshot relationships explicitly and early in the clustering process, while the existing approaches do so implicitly and late in the process. We provide evidence that some dynamic networks need to be partitioned based on topological similarity, and others based on denseness combined with topological similarity, which confirms the need for our approach.

In a comprehensive evaluation, we confirm that ClueNet is able to outperform the existing DNC methods in terms of accuracy on both social and biological datasets a vast majority of the time, either on its own or when combined with the existing methods.

As the availability of temporal network data increases, computational improvements for analyzing (including clustering) such data will only continue to gain importance. This includes further improvements of our ClueNet approach that could potentially allow for even more accurate or faster topological similarity-based clustering of temporal network data. For example, in this study, we have assumed the traditional mathematical definition of a partition of a network, which requires a given node to belong to exactly one cluster. Our work could be extended to allow for overlapping clusters as well, which would be beneficial whenever nodes in a real-world network can belong to multiple functional modules, i.e., can have multiple labels. As another example, our study has dealt with approaches that use only network topology to produce clusters and then use metadata to evaluate the clusters. Clustering approaches exist that integrate network topological information with metadata prior to producing clusters [39–41]. Our work could be extended in this direction as well.

## Supporting information

S1 Section Definition of dynamic graphlets. (PDF)

S2 Section The *k*-medoids algorithm. (PDF)

S3 Section Static network clustering methods extended to DNC. (PDF)

S4 Section Existing DNC method. Multistep [21] is a DNC approach that takes into consideration all snapshots at once when generating a partition (because it was directly proposed in the dynamic setting). Specifically, Multistep does this by modifying the Louvain method to look at the average modularity gain across all snapshots when deciding whether two clusters should be merged. The implementation of Multistep that we use can be found at http://jlguillaume.free.fr/www/programs.php.
(PDF)

S1 Table Node labels for the Enron network. The percentage of nodes in the network that have the given label.
(PDF)

S2 Table Node labels for the hospital network. The percentage of nodes in the network that have the given label.
(PDF)

S3 Table Node labels for the high school network. The percentage of nodes in the network that have the given label.
(PDF)

S4 Table The network construction parameters used by each method. The “method” column displays the clustering algorithm (*k*-medoids or an existing denseness-based method) used by the given version of ClueNet.
(PDF)

S5 Table Results when ClueNet’s dynamic graphlet-based topological similarities are used on top of the existing denseness-based methods. Partition quality results of the given denseness-based methods, ClueNet, and when combining ClueNet’s graphlet-based topological similarities with the denseness-based methods, for the three versions of ClueNet, for the high school and hospital datasets.
(PDF)

S1 Fig Pairwise edge overlaps between the snapshots of social (a) hospital and (b) high school dynamic networks. The darker the color, the higher the edge overlap between the given snapshots. For the hospital data, the following network construction parameter values are used: *t*_*w*_ = 300 seconds and *w* = 1. For the high school data, the parameter values are: *t*_*w*_ = 200 seconds and *w* = 1.
(PDF)

S2 Fig The ranking of the three versions of ClueNet. The ranking of the three versions of ClueNet (C-ST, C-D, and C-C) over all considered **(a)** social datasets (i.e., the three ground truth partitions corresponding to the three social dynamic networks) and **(b)** biological datasets (i.e., the four ground truth partitions corresponding to the biological aging-related dynamic network) with respect to all of precision, recall, and AMI (F-score is excluded here because it is redundant to precision and recall). The ranking is expressed as a percentage of all cases (across all ground truth partitions and all three partition quality measures) in which the given ClueNet version yields the *k*^*th*^ best score across all methods (corresponding to rank *k*). We rank the methods based on their *p*-values (i.e., the smaller the *p*-value, the better the method); in case of ties, we compare the methods based on their raw partition quality scores. The ‘N/A’ rank signifies that the given method did not produce a statistically significant partition under the given partition quality score.
(PDF)

S3 Fig Detailed method comparison in terms of precision. Detailed method comparison results in terms of precision, quantifying how well each method (ClueNet (its three versions: C-ST, C-D, and C-C), Louvain (L), Infomap (I), Hierarchical Infomap (HI), label propagation (LP), simulated annealing (SA), and Multistep (M)) captures each of the three ground truth partitions from the social network domain (top) and each of the four ground truth partitions from the biological network domain (bottom) when clustering the corresponding networks. For each dataset, for each method, we compare the score of the partition produced by the given method (red) to the average score of its random counterparts (blue) and show the resulting *p*-value (see Section Measuring partition quality for details). Then, it is the methods’ *p*-values that should be compared rather than just the raw scores. See S4 Table to see which clustering algorithm the given version of ClueNet uses for the given social dataset.
(PDF)

S4 Fig Detailed method comparison in terms of recall. Detailed method comparison results in terms of recall, quantifying how well each method (ClueNet (its three versions: C-ST, C-D, and C-C), Louvain (L), Infomap (I), Hierarchical Infomap (HI), label propagation (LP), simulated annealing (SA), and Multistep (M)) captures each of the three ground truth partitions from the social network domain (top) and each of the four ground truth partitions from the biological network domain (bottom) when clustering the corresponding networks. For each dataset, for each method, we compare the score of the partition produced by the given method (red) to the average score of its random counterparts (blue) and show the resulting *p*-value (see Section Measuring partition quality for details). Then, it is the methods’ *p*-values that should be compared rather than just the raw scores. See S4 Table to see which clustering algorithm the given version of ClueNet uses for the given social dataset.
(PDF)

S5 Fig Detailed method comparison in terms of F-score. Detailed method comparison results in terms of F-score, quantifying how well each method (ClueNet (its three versions: C-ST, C-D, and C-C), Louvain (L), Infomap (I), Hierarchical Infomap (HI), label propagation (LP), simulated annealing (SA), and Multistep (M)) captures each of the three ground truth partitions from the social network domain (top) and each of the four ground truth partitions from the biological network domain (bottom) when clustering the corresponding networks. For each dataset, for each method, we compare the score of the partition produced by the given method (red) to the average score of its random counterparts (blue) and show the resulting *p*-value (see Section Measuring partition quality for details). Then, it is the methods’ *p*-values that should be compared rather than just the raw scores. See S4 Table to see which clustering algorithm the given version of ClueNet uses for the given social dataset.
(PDF)

S6 Fig Detailed method comparison in terms of AMI. Detailed method comparison results in terms of AMI, quantifying how well each method (ClueNet (its three versions: C-ST, C-D, and C-C), Louvain (L), Infomap (I), Hierarchical Infomap (HI), label propagation (LP), simulated annealing (SA), and Multistep (M)) captures each of the three ground truth partitions from the social network domain (top) and each of the four ground truth partitions from the biological network domain (bottom) when clustering the corresponding networks. For each dataset, for each method, we compare the score of the partition produced by the given method (red) to the average score of its random counterparts (blue) and show the resulting *p*-value (see Section Measuring partition quality for details). Then, it is the methods’ *p*-values that should be compared rather than just the raw scores. See S4 Table to see which clustering algorithm the given version of ClueNet uses for the given social dataset.
(PDF)

S7 Fig Pairwise partition similarities in terms of AMI. Pairwise similarities in terms of AMI between different partitions (ground truth (GT), ClueNet (its three versions: C-ST, C-D, and C-C), Louvain (L), Infomap (I), Hierarchical Infomap (HI), label propagation (LP), simulated annealing (SA), and Multistep (M)), for **(a)** social Enron, **(b)** social hospital, **(c)** social high school, and **(d)** biological aging-related dynamic networks. Note that in panel (d), there are four ground truth partitions, depending on which aging-related ground truth data is considered (BE2004, BE2008, AD, or SequenceAge, which are labeled as GT1, GT2, GT3, and GT4 respectively; Section Data).
(PDF)

S8 Fig Running time comparison. Running time comparison of the different methods (ClueNet (its three versions: C-ST, C-D, C-C), Louvain (L), Infomap (I), Hierarchical Infomap (HI), label propagation (LP), simulated annealing (SA), and Multistep (M)) for the social **(a)** hospital and **(b)** high school dynamic networks.
(PDF)
