# Supplementary material for: ClueNet: Clustering a temporal network based on topological similarity rather than denseness
Source: PLoS One. 2018 May 8;13(5):e0195993. doi: 10.1371/journal.pone.0195993 (PMC5940177; doi:10.1371/journal.pone.0195993)
Supplement: S1 Section — (PDF) [file pone.0195993.s001.pdf]

S1 Section. Definition of dynamic graphlets. To more formally define dynamic graphlets, we must first introduce the notion of a Δt-time-respecting path (in the raw temporal network dataset $D(V, E)$ that contains information on duration of each event). A Δt-time-respecting path between two nodes is a sequence of events that connects the two nodes such that for any two consecutive events in the sequence, the start time of the later event and the end time of the earlier event are within Δt time of each other. A temporal dataset $D(V, E)$ is then considered Δt-connected if there exists a Δt-time-respecting path between every pair of nodes in the data. If $D'(V', E')$ is a temporal subgraph of D with $V' \subseteq V$ and $E' \subseteq E$ (where E' is restricted to nodes in V'), then a dynamic graphlet is an equivalence class of isomorphic Δt-connected subgraphs; two dynamic graphlets are equivalent if they both have the same relative order of events, regardless of the events' start times.

When considering datasets that are snapshots-based, meaning that event duration is not provided, we consider each edge between any pair of nodes from snapshot i of the given dataset to be an event that is active from time i to time $i + 1$. Then, all of the above definitions that rely on event durations are applicable to the datasets.
