# Supplementary material for: ClueNet: Clustering a temporal network based on topological similarity rather than denseness
Source: PLoS One. 2018 May 8;13(5):e0195993. doi: 10.1371/journal.pone.0195993 (PMC5940177; doi:10.1371/journal.pone.0195993)
Supplement: S2 Section — (PDF) [file pone.0195993.s002.pdf]

S2 Section. The k-medoids algorithm. In this study, we use a python implementation of the k-medoids algorithm, which is a modification of the classic k-means algorithm that chooses actual data points (in our case, nodes) as centers, i.e., medoids; a medoid is the "central" data point of a cluster whose average distance to all other data points in the cluster is minimal. The k-medoids algorithm steps are as follows: (1) pick k nodes as centers of k clusters and assign all the remaining nodes in the dynamic network to these centers; each node will be assigned to the center that has minimal distance (i.e., maximal similarity) to it; solve any ties randomly; (2) in each cluster i, pick node v as the new center of the cluster, so as to minimize the total sum of distances between node v and all other nodes in cluster i; (3) reassign all nodes to the new centers, as explained in step (1); and (4) repeat the previous two steps until the algorithm converges, i.e., until the same set of centers are being chosen from one iteration to the next.
