# Supplementary material for: ClueNet: Clustering a temporal network based on topological similarity rather than denseness
Source: PLoS One. 2018 May 8;13(5):e0195993. doi: 10.1371/journal.pone.0195993 (PMC5940177; doi:10.1371/journal.pone.0195993)
Supplement: S3 Section — (PDF) [file pone.0195993.s003.pdf]

S3 Section. Static network clustering methods extended to DNC. Several static network clustering methods have been adjusted to perform DNC [10]: Louvain [16], Infomap [17], Hierarchical Infomap [18], label propagation [19], and simulated annealing [20]. Before going into how these methods were transformed into their DNC versions, we first give a brief overview of how (the static version of) each method works, noting that for each method, the input is a static network and the output is a single partition.

The Louvain method initially treats each node as its own cluster. If there is an increase in modularity when merging a cluster to another cluster that is adjacent to it in the network, then the clusters are merged (modularity is the fraction of intra-cluster edges out of all edges in the actual network minus the same fraction if edges were distributed at random, and so, a network will have high modularity if it has significantly dense interconnectivity between nodes within clusters but sparse interconnectivity between nodes in different clusters). This process is repeated until modularity can no longer be increased. Next, a new network is built in which nodes are the clusters resulting from the iterative merging procedure, and the iterative merging procedure is applied on the new network. Then, the entire process of building a new network and applying the iterative cluster merging procedure on the new network is repeated until modularity can no longer be improved.

Infomap treats clustering like a data compression problem (whose goal is to use the least amount of data to represent something). Essentially, Infomap aims to find the smallest amount of bits needed to describe a random walk across every node in a network. By assigning a name to each node (using bits), one can describe a random walk across every node in a network simply by storing the name of node that the walker traverses at each step. However, this process results in using a lot of bits. Infomap reduces the amount of bits needed to describe a walk across every node in a network by first identifying clusters using the random walk (a random walker is statistically likely to spend long periods of time within certain clusters of nodes). The algorithm then assigns each cluster a unique name. Thus, when the walker enters a cluster, Infomap first stores the name of the cluster the walker enters, then lists the nodes the walker traversed. Now that it begins by listing the cluster it is in, Infomap can re-label the nodes in the network such that a node's name only has to be unique within its own cluster (i.e., nodes in different clusters can have the same name); hence, the name of each node can be shorter, which consequently results in fewer bits needed to describe the path of the walker.

Hierarchical Infomap is essentially a modification of the Louvain method. In the Louvain method, once clusters are merged, they remain merged (i.e., cluster memberships cannot change)

for the remainder of the algorithm's execution. Thus, what was once an optimal move during the early stages of an algorithm may actually hurt the algorithm during its later stages. Hierarchical Infomap aims to account for this by allowing nodes to change cluster memberships after the final step of the Louvain method. Hierarchical Infomap treats each cluster as its own network and partitions each of these networks into one or more clusters, thus creating a new partition of the entire (original) network. The merging process of the Louvain method is then reapplied on the new partition of the entire network.

Label propagation assigns to each node a unique label, and then it iteratively re-assigns to each node u the label that the majority of u's neighbors have, until the algorithm converges.

Simulated annealing begins by generating a random partition. It then randomly changes the cluster membership of a randomly selected node. If the resulting partition shows an increase in modularity, then it is declared as the new (i.e. more optimal) partition; otherwise, this partition is considered to be the new partition with some probability p (i.e., the original partition is kept as the new partition with probability 1-p) to avoid being trapped in a local minimum. This process is repeated until a designated stopping point is reached.

The implementations of the DNC versions of these methods that we use in this study can be found at `https://sites.google.com/site/andrealancichinetti/software`.
