# Supplementary material for: ClueNet: Clustering a temporal network based on topological similarity rather than denseness
Source: PLoS One. 2018 May 8;13(5):e0195993. doi: 10.1371/journal.pone.0195993 (PMC5940177; doi:10.1371/journal.pone.0195993)
Supplement: S4 Section — Multistep [21] is a DNC approach that takes into consideration all snapshots at once when generating a partition (because it was directly proposed in the dynamic setting). Specifically, Multistep does this by modifying the Louvain method to look at the average modularity gain across all snapshots when deciding whether two clusters should be merged. The implementation of Multistep that we use can be found at http://jlguillaume.free.fr/www/programs.php. (PDF) [file pone.0195993.s004.pdf]

S4 Section. Existing DNC method. Multistep is a DNC approach that takes into consideration all snapshots at once when generating a partition (because it was directly proposed in the dynamic setting). Specifically, Multistep does this by modifying the Louvain method to look at the average modularity gain across all snapshots when deciding whether two clusters should be merged. The implementation of Multistep that we use can be found at `http://jlguillaume.free.fr/www/programs.php`.
