# Supplementary material for: ClueNet: Clustering a temporal network based on topological similarity rather than denseness
Source: PLoS One. 2018 May 8;13(5):e0195993. doi: 10.1371/journal.pone.0195993 (PMC5940177; doi:10.1371/journal.pone.0195993)
Supplement: S1 Table — The percentage of nodes in the network that have the given label. (PDF) [file pone.0195993.s005.pdf]

Table S1. Node labels for the Enron network.

**Label**	**Employee**	**VP**	**Manager**	**CEO**	**Director**	**President**	**Managing Director**
**Percentage**	58.2%	16.5%	8.8%	2.7%	7.7%	2.7%	3.3%

The percentage of nodes in the network that have the given label.
