# Supplementary material for: ClueNet: Clustering a temporal network based on topological similarity rather than denseness
Source: PLoS One. 2018 May 8;13(5):e0195993. doi: 10.1371/journal.pone.0195993 (PMC5940177; doi:10.1371/journal.pone.0195993)
Supplement: S2 Table — The percentage of nodes in the network that have the given label. (PDF) [file pone.0195993.s006.pdf]

Table S2. Node labels for the hospital network.

**Label**	**Doctor**	**Administrator**	**Nurse**	**Patient**
**Percentage**	14.7%	10.7%	36.0%	38.7%

The percentage of nodes in the network that have the given label.
