# Supplementary material for: ClueNet: Clustering a temporal network based on topological similarity rather than denseness
Source: PLoS One. 2018 May 8;13(5):e0195993. doi: 10.1371/journal.pone.0195993 (PMC5940177; doi:10.1371/journal.pone.0195993)
Supplement: S3 Table — The percentage of nodes in the network that have the given label. (PDF) [file pone.0195993.s007.pdf]

Table S3. Node labels for the high school network.

**Label**	**2BIO1**	**2BIO2**	**2BIO3**	**PC**	**PC***	**PSI***	**MP**	**MP*1**	**MP*2**
**Percentage**	11.3%	10.0%	12.2%	13.5%	12.0%	10.4%	10.1%	8.9%	11.6%

The percentage of nodes in the network that have the given label.
