# Supplementary material for: ClueNet: Clustering a temporal network based on topological similarity rather than denseness
Source: PLoS One. 2018 May 8;13(5):e0195993. doi: 10.1371/journal.pone.0195993 (PMC5940177; doi:10.1371/journal.pone.0195993)
Supplement: S4 Table — The “method” column displays the clustering algorithm (k-medoids or an existing denseness-based method) used by the given version of ClueNet. (PDF) [file pone.0195993.s008.pdf]

Table S4. The network construction parameters used by each method

	Enron			Hospital			High school		
	tw	w	Method	tw	w	Method	tw	w	Method
**C-ST**	3 months	2	K-Means	500	16	Label Propagation	200	1	Simulated Annealing
**C-D**	3 months	8	K-Means	50	2	Hierarchical Infomap	100	1	Simulaed Annealing
**C-C**	2 months	2	K-Means	50	1	K-Means	200	1	Simulated Annealing
**Louvain**	1 month	2	-	-	-	-	200	16	-
**Infomap**	2 weeks	8	-	300	2	-	200	16	-
**Hierarchical Infomap**	2 weeks	8	-	300	2	-	200	2	-
**Label Propagation**	3 months	16	-	300	8	-	200	16	-
**Simulated Annealing**	1 month	16	-	100	1	-	200	1	-
**Multistep**	2 months	2	-	300	8	-	100	1	-

The “method” column displays the clustering algorithm (k-medoids or an existing denseness-based method) used by the given version of ClueNet.
