# Supplementary material for: ClueNet: Clustering a temporal network based on topological similarity rather than denseness
Source: PLoS One. 2018 May 8;13(5):e0195993. doi: 10.1371/journal.pone.0195993 (PMC5940177; doi:10.1371/journal.pone.0195993)
Supplement: S5 Table — Partition quality results of the given denseness-based methods, ClueNet, and when combining ClueNet’s graphlet-based topological similarities with the denseness-based methods, for the three versions of ClueNet, for the high school and hospital datasets. (PDF) [file pone.0195993.s009.pdf]

Table S5. Results when ClueNet's dynamic graphlet-based topological similarities are used on top of the existing denseness-based methods.

C-ST

Method	Precision	Recall	AMI	
Simulated Annealing	96.68%	93.89%	0.9372	High School
ClueNet	17.10%	35.98%	0.1752	
Combined	99.44%	99.39%	0.9933	
Label Propagation	42.62%	90.55%	0.2851	Hospital
ClueNet	45.15%	45.48%	0.2249	
Combined	43.32%	65.26%	0.258	

C-D

Method	Precision	Recall	AMI	
Simulated Annealing	96.68%	93.89%	0.9372	High School
ClueNet	14.73%	37.27%	0.1467	
Combined	99.44%	99.39%	0.9933	
Hi. Infomap	43.79%	91.57%	0.3389	Hospital
ClueNet	50.96%	69.40%	0.2698	
Combined	42.91%	69.88%	0.3528	

C-C

Method	Precision	Recall	AMI	
Simulated Annealing	96.68%	93.89%	0.9372	High School
ClueNet	12.25%	62.40%	0.0795	
Combined	99.44%	99.39%	0.9933	

Partition quality results of the given denseness-based methods, ClueNet, and when combining ClueNet's graphlet-based topological similarities with denseness-based methods, for the three versions of ClueNet, for the high school and hospital datasets.
