# Supplementary material for: ClueNet: Clustering a temporal network based on topological similarity rather than denseness
Source: PLoS One. 2018 May 8;13(5):e0195993. doi: 10.1371/journal.pone.0195993 (PMC5940177; doi:10.1371/journal.pone.0195993)
Supplement: S1 Fig — The darker the color, the higher the edge overlap between the given snapshots. For the hospital data, the following network construction parameter values are used: tw = 300 seconds and w = 1. For the high school data, the parameter values are: tw = 200 seconds and w = 1. (PDF) [file pone.0195993.s010.pdf]

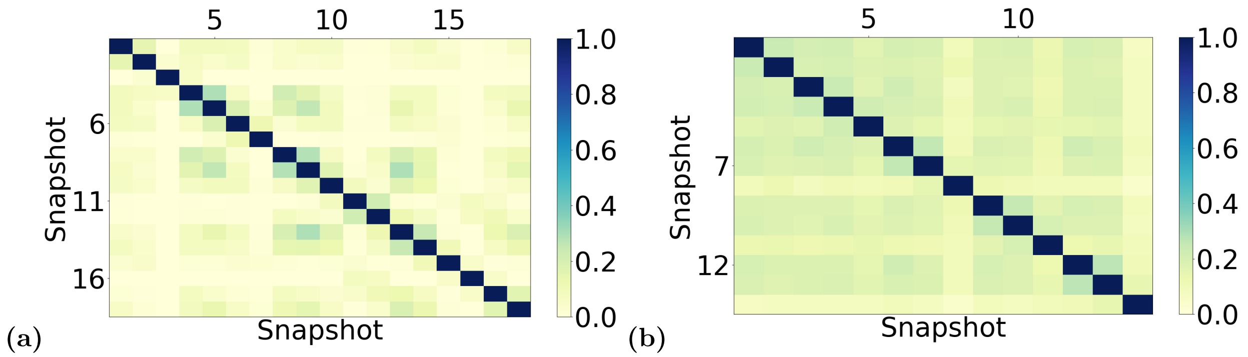

Fig S1. Pairwise edge overlaps between the snapshots of social (a) hospital and (b) high school dynamic networks. The darker the color, the higher the edge overlap between the given snapshots. For the hospital data, the following network construction parameter values are used: t_w=300 seconds and w=1. For the high school data, the parameter values are: t_w=200 seconds and w=1.
