# Supplementary material for: ClueNet: Clustering a temporal network based on topological similarity rather than denseness
Source: PLoS One. 2018 May 8;13(5):e0195993. doi: 10.1371/journal.pone.0195993 (PMC5940177; doi:10.1371/journal.pone.0195993)
Supplement: S2 Fig — The ranking of the three versions of ClueNet (C-ST, C-D, and C-C) over all considered (a) social datasets (i.e., the three ground truth partitions corresponding to the three social dynamic networks) and (b) biological datasets (i.e., the four ground truth partitions corresponding to the biological aging-related dynamic network) with respect to all of precision, recall, and AMI (F-score is excluded here because it is redundant to precision and recall). The ranking is expressed as a percentage of all cases (across all ground truth partitions and all three partition quality measures) in which the given ClueNet version yields the kth best score across all methods (corresponding to rank k). We rank the methods based on their p-values (i.e., the smaller the p-value, the better the method); in case of ties, we compare the methods based on their raw partition quality scores. The ‘N/A’ rank signifies that the given method did not produce a statistically significant partition under the given partition quality score. (PDF) [file pone.0195993.s011.pdf]

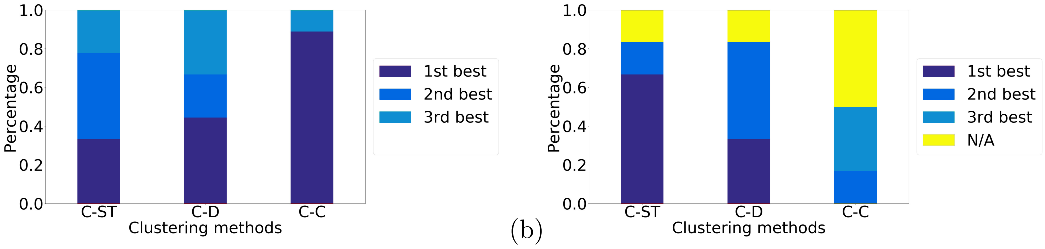

(a) (b)

Fig S2. The ranking of the three versions of ClueNet. The ranking of the three versions of ClueNet (C-ST, C-D, and C-C) over all considered **(a)** social datasets (i.e., the three ground truth partitions corresponding to the three social dynamic networks) and **(b)** biological datasets (i.e., the four ground truth partitions corresponding to the biological aging-related dynamic network) with respect to all of precision, recall, and AMI (F-score is excluded here because it is redundant to precision and recall). The ranking is expressed as a percentage of all cases (across all ground truth partitions and all three partition quality measures) in which the given ClueNet version yields the kth best score across all methods (corresponding to rank k). We rank the methods based on their p-values (i.e., the smaller the p-value, the better the method); in case of ties, we compare the methods based on their raw partition quality scores. The 'N/A' rank signifies that the given method did not produce a statistically significant partition under the given partition quality score.
