# Supplementary material for: ClueNet: Clustering a temporal network based on topological similarity rather than denseness
Source: PLoS One. 2018 May 8;13(5):e0195993. doi: 10.1371/journal.pone.0195993 (PMC5940177; doi:10.1371/journal.pone.0195993)
Supplement: S5 Fig — Detailed method comparison results in terms of F-score, quantifying how well each method (ClueNet (its three versions: C-ST, C-D, and C-C), Louvain (L), Infomap (I), Hierarchical Infomap (HI), label propagation (LP), simulated annealing (SA), and Multistep (M)) captures each of the three ground truth partitions from the social network domain (top) and each of the four ground truth partitions from the biological network domain (bottom) when clustering the corresponding networks. For each dataset, for each method, we compare the score of the partition produced by the given method (red) to the average score of its random counterparts (blue) and show the resulting p-value (see Section Measuring partition quality for details). Then, it is the methods’ p-values that should be compared rather than just the raw scores. See S4 Table to see which clustering algorithm the given version of ClueNet uses for the given social dataset. (PDF) [file pone.0195993.s014.pdf]

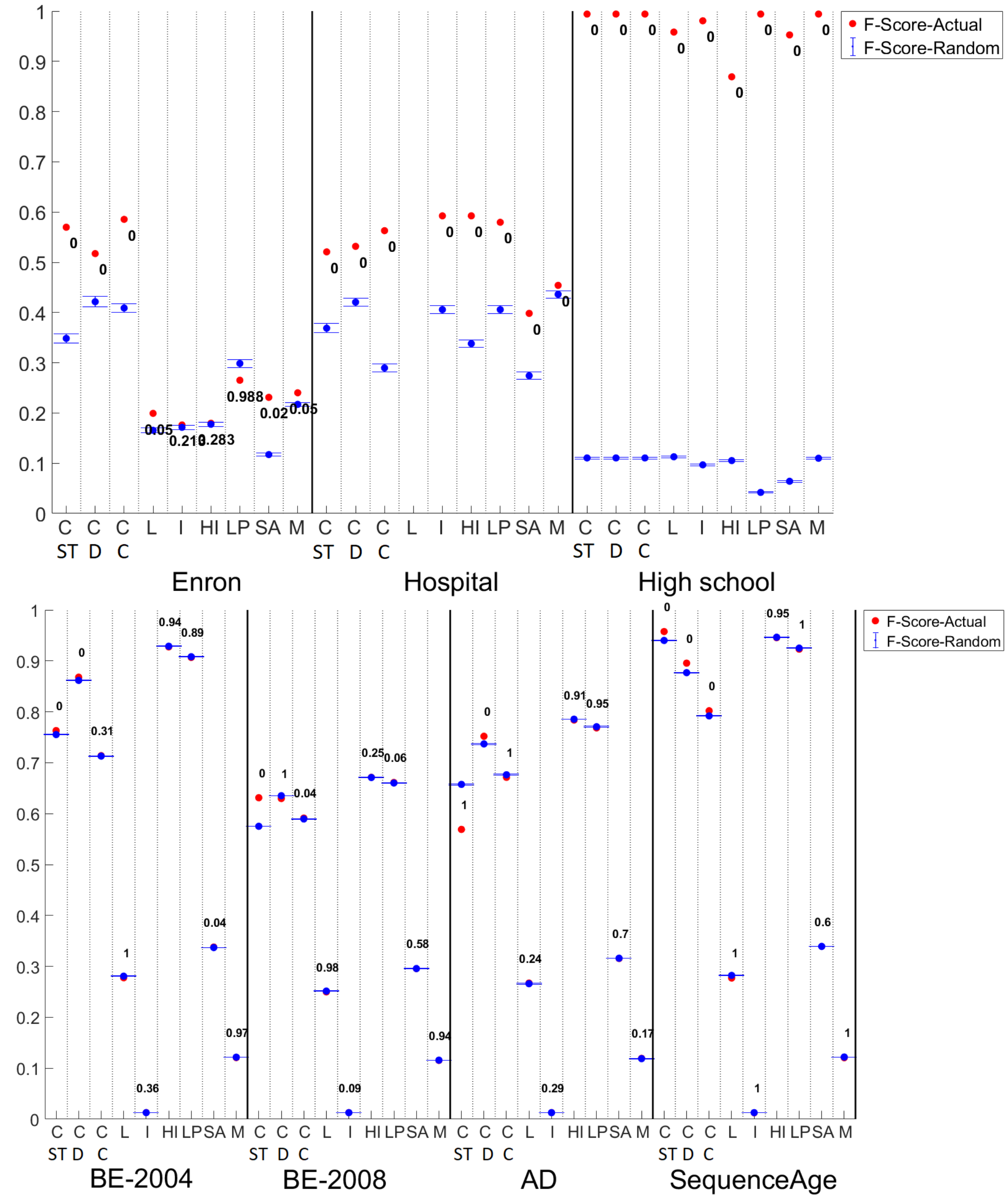

Fig S5. Detailed method comparison in terms of F-score. Detailed method comparison results in terms of F-score, quantifying how well each method (ClueNet (its three versions: C-ST, C-D, and C-C), Louvain (L), Infomap (I), Hierarchical Infomap (HI), label propagation (LP), simulated annealing (SA), and Multistep (M)) captures each of the three ground truth partitions from the social network domain (top) and each of the four ground truth partitions from the biological network domain (bottom) when clustering the corresponding networks. For each dataset, for each method, we compare the score of the partition produced by the given method (red) to the average score of its random counterparts (blue) and show the resulting p-value (see Section Measuring partition quality for details). Then, it is the methods' p-values that should be compared rather than just the raw scores. See Table S4 to see which clustering algorithm the given version of ClueNet uses for the given social dataset.
