# Supplementary material for: ClueNet: Clustering a temporal network based on topological similarity rather than denseness
Source: PLoS One. 2018 May 8;13(5):e0195993. doi: 10.1371/journal.pone.0195993 (PMC5940177; doi:10.1371/journal.pone.0195993)
Supplement: S7 Fig — Pairwise similarities in terms of AMI between different partitions (ground truth (GT), ClueNet (its three versions: C-ST, C-D, and C-C), Louvain (L), Infomap (I), Hierarchical Infomap (HI), label propagation (LP), simulated annealing (SA), and Multistep (M)), for (a) social Enron, (b) social hospital, (c) social high school, and (d) biological aging-related dynamic networks. Note that in panel (d), there are four ground truth partitions, depending on which aging-related ground truth data is considered (BE2004, BE2008, AD, or SequenceAge, which are labeled as GT1, GT2, GT3, and GT4 respectively; Section Data). (PDF) [file pone.0195993.s016.pdf]

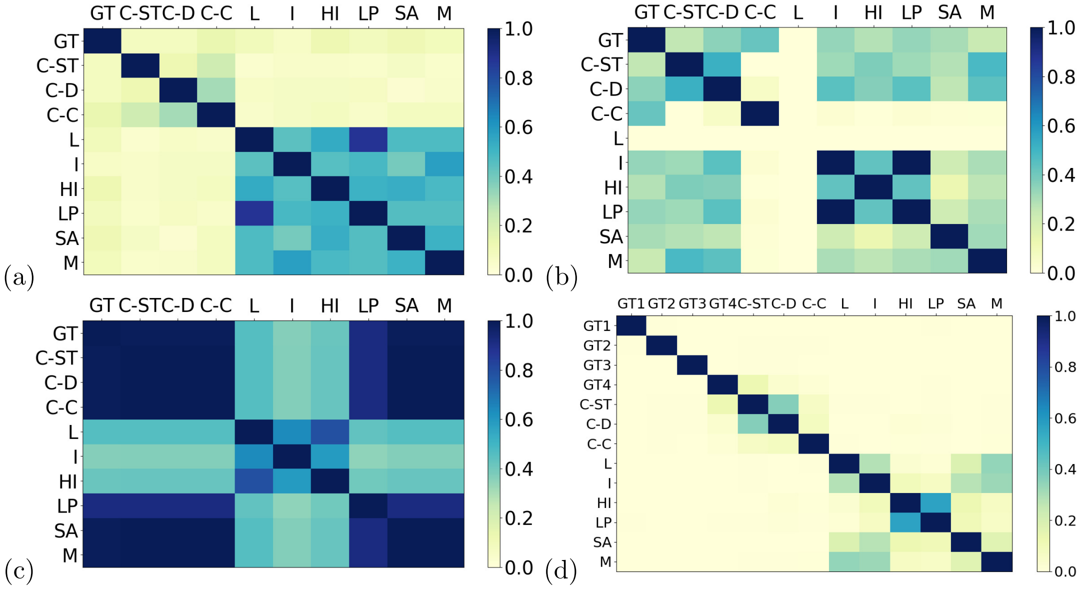

Fig S7. Pairwise partition similarities in terms of AMI. Pairwise similarities in terms of AMI between different partitions (ground truth (GT), ClueNet (its three versions: C-ST, C-D, and C-C), Louvain (L), Infomap (I), Hierarchical Infomap (HI), label propagation (LP), simulated annealing (SA), and Multistep (M)), for **(a)** social Enron, **(b)** social hospital, **(c)** social high school, and **(d)** biological aging-related dynamic networks. Note that in panel (d), there are four ground truth partitions, depending on which aging-related ground truth data is considered (BE2004, BE2008, AD, or SequenceAge, which are labeled as GT1, GT2, GT3, and GT4 respectively; Section Data.
