# Supplementary material for: ClueNet: Clustering a temporal network based on topological similarity rather than denseness
Source: PLoS One. 2018 May 8;13(5):e0195993. doi: 10.1371/journal.pone.0195993 (PMC5940177; doi:10.1371/journal.pone.0195993)
Supplement: S8 Fig — Running time comparison of the different methods (ClueNet (its three versions: C-ST, C-D, C-C), Louvain (L), Infomap (I), Hierarchical Infomap (HI), label propagation (LP), simulated annealing (SA), and Multistep (M)) for the social (a) hospital and (b) high school dynamic networks. (PDF) [file pone.0195993.s017.pdf]

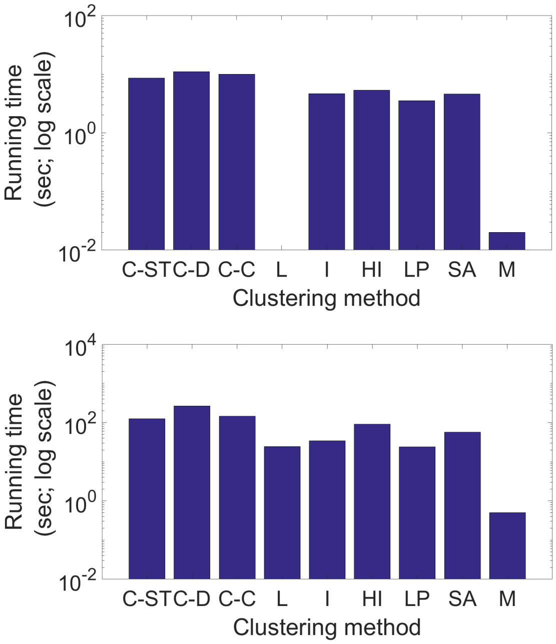

(a)

(b)

Fig S8. Running time comparison. Running time comparison of the different methods (ClueNet (its three versions: C-ST, C-D, C-C), Louvain (L), Infomap (I), Hierarchical Infomap (HI), label propagation (LP), simulated annealing (SA), and Multistep (M)) for the social **(a)** hospital and **(b)** high school dynamic networks.
